# Hypotension caused by oral administration of 5-aminolevulinic acid persists after surgery in patients undergoing transurethral resection of bladder tumor under spinal anesthesia

**DOI:** 10.1186/s40981-020-00399-4

**Published:** 2020-11-21

**Authors:** Masami Sato, Tsutomu Yanagisawa, Sonoko Minamino, Toshiyuki Arai

**Affiliations:** Department of Anesthesia, Kyoto City Hospital, 1-2 Mibuhigashitakada-cho, Nakagyo-ku, Kyoto, 604-8845 Japan

To the Editor,

5-Aminolevulinic acid (ALA) is utilized for photodynamic diagnosis to detect superficial bladder cancer and its adverse effect is hypotension [1–5]. Compared to general anesthesia, spinal anesthesia is not indicated as an increased risk factor for 5-ALA-induced hypotension from anesthesia induction to the start of surgery in patients undergoing transurethral resection of bladder tumor (TUR-BT) with 5-ALA [2, 3]. However, no studies exist that have investigated the precise perioperative hypotensive effects by 5-ALA under spinal anesthesia. This retrospective study assessed the hypotensive effect of 5-ALA on hemodynamic change of spinal anesthesia in patients undergoing TUR-BT not only during surgery but also after the ward admission.

The medical records of 129 patients who underwent TUR-BT under spinal anesthesia using 0.5% hyperbaric bupivacaine in Kyoto City Hospital between January 2018 and March 2019 were divided into two groups, based on whether 5-ALA was used (ALA group, *n* = 66) or not used (control group, *n* = 63). The ALA group received 5-ALA (20 mg/kg) orally 3 h before the start of surgery. The change in noninvasive blood pressure (BP) on the day of surgery and the dosage of vasopressors used during spinal anesthesia were analyzed.

No significant difference existed between the two groups in the patients’ preoperative characteristics. Intravenous fluid volume during anesthesia was significantly higher in the ALA group than in the control group (Table 1). Systolic and diastolic BP were significantly lower in the ALA group than in the control group from spinal anesthesia until 2 h after the ward admission. In the ALA group, systolic and diastolic BP significantly remained decreased from spinal anesthesia until 2 h after the ward admission (Fig. 1). The total dosage of ephedrine and phenylephrine were higher in the ALA group than in the control group (Table 1).

**Table 1:** The patients’ preoperative characteristics, anesthesia profiles and the drugs administered during anesthesia

	Control group (*n* = 63)	ALA group (*n* = 66)	*P* value
Age (years)	71 ± 11	74 ± 10	0.27
Height (cm)	162 ± 15	161 ± 14	0.58
Weight (kg)	65 ± 17	65 ± 18	0.97
Sex (male/female)	59 (94%)/4 (6%)	55 (83%)/11 (17%)	0.07
Regular use of antihypertensive drug	24 (38%)	23 (35%)	0.70
Regular use of renin-angiotensin system inhibitor	14 (22%)	11 (17%)	0.42
Duration of surgery (minutes)	43 ± 25	44 ± 20	0.84
Duration of anesthesia (minutes)	75 ± 28	80 ± 22	0.25
Dose of 0.5% hyperbaric bupivacaine of spinal anesthesia (mL)	3.1 ± 0.3	3.0 ± 0.3	0.20
Sensory block level just before surgery^a^	T8 (T2–T11)	T6 (T2–L1)	0.26
Sensory block level at the end of surgery^a^	T6 (T2–T12)	T5 (T2–T12)	0.49
Intravenous fluid volume during anesthesia (mL)	570 ± 191	758 ± 284	< 0.001
Use of vasopressors^b^	10 (16%)	47 (71%)	< 0.001
Use of ephedrine	8 (13%)	37 (56%)	< 0.001
Total dose of ephedrine (mg)	1.0 ± 3.0	6.9 ± 9.0	< 0.001
Use of phenylephrine	6 (10%)	30 (45%)	< 0.001
Total dose of phenylephrine (mg)	0.013 ± 0.042	0.21 ± 0.34	< 0.001

Values are presented as mean ± standard deviation or number (%)

^a^Median (maximum-minimum)

^b^Ephedrine or phenylephrine or both were used

*ALA* aminolevulinic acid

**Fig. 1:**
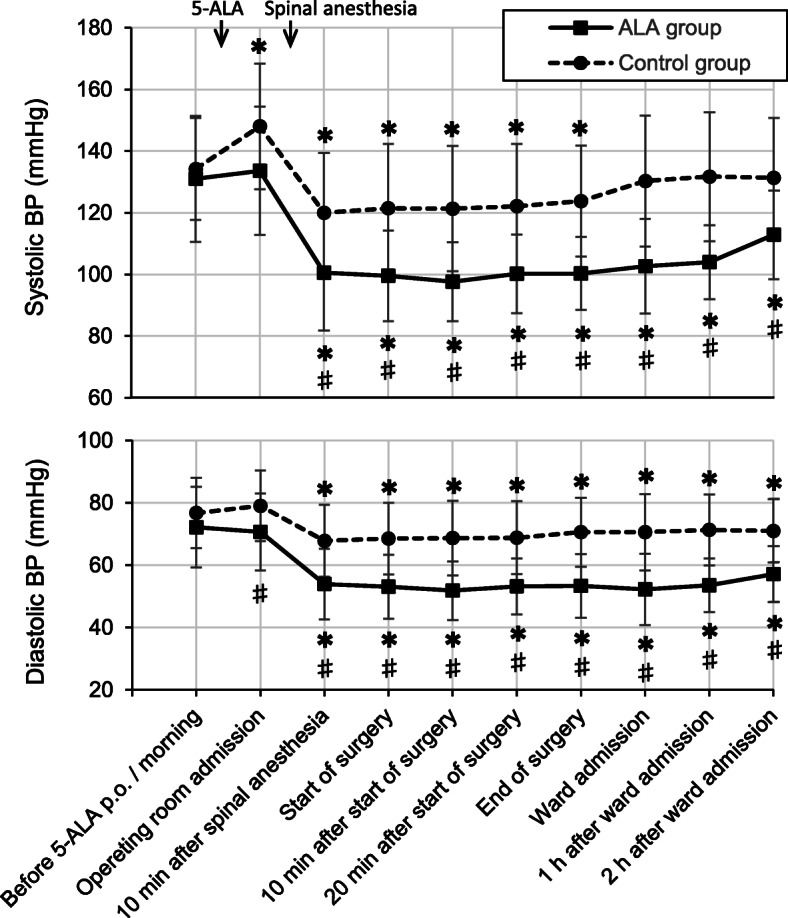
Time courses of systolic blood pressure and diastolic blood pressure. Values are presented as mean ± standard deviation. *P < 0.05, post-operating room admission versus before 5-ALA p.o./morning. ^#^*P* < 0.05, the ALA group versus the control group. *ALA* aminolevulinic acid, *BP* blood pressure, *p.o.* per os

Additive hypotensive effect induced by spinal anesthesia and 5-ALA was prominent after spinal anesthesia induction not only during surgery but also for 2 h after the ward admission, as shown by the BP changes in our ALA group. Since the duration of the hypotensive effect by 5-ALA is longer than that of hyperbaric bupivacaine of spinal anesthesia, the systolic BP in the ALA group did not return to the baseline level even 2 h after the ward admission, unlike in the control group. We cannot easily state that spinal anesthesia using hyperbaric bupivacaine is a safe technique from our result, when considering the postoperative period. Our reflection point is that hypotension may be encouraged by hypovolemia in the ALA group, because all patients did not have intravenous fluids after 5-ALA administration before spinal anesthesia. Other risk factors that increase the incidence of 5-ALA-induced hypotension have been reported to be the regular use of antihypertensive drugs, particularly renin-angiotensin system inhibitor drugs [2, 4] and a history of ALA-induced hypotension [5].

In conclusion, orally administered 5-ALA before TUR-BT under spinal anesthesia induced significant hypotension after spinal anesthesia induction until 2 h after surgery and an increased need for vasopressors and intravenous volume. Anesthesiologists should have a strategy for safe anesthetic management against long-lasting hypotension by 5-ALA.

## Data Availability

The datasets used and analyzed during the current study are available from the corresponding author on reasonable request.

## Abbreviations

ALA: Aminolevulinic acid
TUR-BT: Transurethral resection of bladder tumor
BP: Blood pressure
